# Editorial: “Novel Pain Therapeutics: From Basic Research to Clinical Translation and Rehabilitation”

**DOI:** 10.3389/fphar.2021.681422

**Published:** 2021-04-14

**Authors:** Damiana Scuteri, Tsukasa Sakurada, Paolo Tonin, Maria Tiziana Corasaniti, Giacinto Bagetta

**Affiliations:** ^1^ Pharmacotechnology Documentation and Transfer Unit, Section of Preclinical and Translational Pharmacology, Department of Pharmacy, Health and Nutritional Sciences, University of Calabria, Rende, Italy; ^2^ Regional Center for Serious Brain Injuries, S. Anna Institute, Crotone, Italy; ^3^ Center for Supporting Pharmaceutical Education, Faculty of Pharmaceutical Sciences, Daiichi University of Pharmacy, Fukuoka, Japan; ^4^ Department of Health Sciences, University “Magna Graecia” of Catanzaro, Catanzaro, Italy; ^5^ School of Hospital Pharmacy, University “Magna Graecia” of Catanzaro, Catanzaro, Italy

**Keywords:** pain, sensitization, natural and synthetic analgesics, opioids, dementia, cognitive impairment, rehabilitation

Chronic pain is a major public health problem representing a global leading cause of disability and disease burden with low back pain and headache disorders ranking top-ten as causes of disability-adjusted life-years (DALYs) throughout the whole lifetime from teenage to old age (Diseases and Injuries, 2020). In fact, some 30–50% of the world population and almost 20% of the Europeans actually suffer from chronic pain with devastating cost for the society in terms of disability and hospitalization (Breivik et al., 2006; Reid et al., 2011; van Hecke et al., 2013; Mills et al., 2019). Therefore, research aiming at understanding the mechanisms involved in chronic pain is the key to improve treatment and quality of life of patients. This is even more important at the time of COVID-19 that poses several limitations to the access to chronic pain treatment (Karos et al., 2020), enhancing its impact especially on aged, cognitively impaired and non communicative patients (Scuteri et al., 2020b). For a better understanding and management of pain there are four main aspects to be considered: 1) neurotransmission and anomalous plasticity; 2) neuron-glial interaction in central sensitization; 3) drugs and botanicals for pain therapy; 4) pharmacology, assessment and management of chronic pain in the fragile populations. Nociceptive primary afferent fibers release mainly glutamate, substance P and calcitonin gene-related peptide (CGRP); modulation of pain by these neurotransmitters is fundamental for translation into clinical practice. Accordingly, understanding of the role of CGRP neurotransmission represents a noteworthy example of advances in migraine prevention and treatment, previously relying only on triptans and symptomatic, non specific, analgesics (Scuteri et al., 2020a). The progress guaranteed by anti-CGRP monoclonal antibodies for migraine, and by eptinezumab, in particular, allowing faster action due to intravenous administration (Scuteri et al., 2019b), is due to the dissection of CGRP signaling mechanisms (Scuteri et al., 2019a). The modulation of the endogenous pain inhibitory system is implicated in the action of pain-relieving drugs (Ossipov et al., 2010). Metabotropic glutamate receptors can promote chronic pain through sensitization of the central nucleus of the amygdala (Ossipov et al., 2010). Thus, glutamatergic transmission is involved in the ascending but also in the descending pathways modulation due to presynaptic glutamate metabotropic autoreceptors like mGlu2/3. The latter are involved in the mechanisms of antinociception of drugs and botanicals (Scuteri et al., 2019d). In fact, the essential oil of bergamot is able to modulate synaptic level of glutamate (Morrone et al., 2007) and this contributes to its analgesic activity (Scuteri et al., 2019c), which is enriched by anxiolytic properties devoid of sedation (Rombola et al., 2019). Interestingly, the analgesic effect occurs also when it is administered via the inhalatory route (Scuteri et al., 2018a), though it is independent on olfactory stimulation and mediated by systemic absorption of active principles (Hamamura et al.). Trazodone has been demonstrated to restore acutely the aberrant plasticity to which mGlu autoreceptors are subjected after spinal nerve ligation (Cisani et al.). Furthermore, glutamate is involved in the antinociceptive effect of apelin, the endogenous ligand for the putative receptor protein related to the type 1 angiotensin receptor (APJ) orphan G-protein-coupled receptor (Lv et al.). The activation of N-methyl-D-aspartate (NMDA) receptor elicits cholecystokinin-8 (CCK-8) pronociceptive effects (Hayashi et al.). Transient Receptor Potential (TRP) Vanilloid 1 and Ankyrin 1 (TRPV1, TRPA1) channels represent important targets for analgesia at level of the nociceptive primary sensory neurons (Horváth et al.). The neuropeptide oxytocin is endowed with preemptive analgesic properties in a validated postoperative pain model (Espinosa De Los Monteros-Zuniga et al.). Galanin receptor agonists have been demonstrated to have potential analgesic properties with action on protein kinase C (Li et al.). Opioid receptors occurring on nociceptor terminals play a pivotal role in pain modulation. For instance, in neuropathic pain deltorphin II can inhibit the response of C fibers in a concentration-dependent and delta opioid receptors-mediated manner (Joukal et al.). Moreover, methadone has been found to be effective in morphine-resistant hyperalgesia and to restore morphine antinociception in the inflammatory model of the complete Freund’s adjuvant (CFA) (Watanabe et al.). The latter is interesting also in view of the lack of efficacy and the onset of serious side effects for chronic administration of opioids in the treatment of osteoarthritis. The role of the inflammasome in pain is fundamental and the interaction between immune and nervous system contributes to the development of chronic pain. Also, the modulation of microglia can reduce allodynia in a sex-dependent manner (Saika et al.). Oxidative stress and nuclear factor erythroid derived-2-related factor 2 (Nrf2) pathway is notably implicated in neuropathic pain (Zhao et al.). Other targets for pain treatment are the small-conductance Ca^2+^-activated K^+^ channels for visceral hypersensitivity (Ji et al.). In particular, the study of visceral hypersensitivity is of the utmost importance since it can predispose to visceral chronic pain and it can be caused by maternal separation responsible for elevated firing frequency of corticotropin-releasing factor neurons (Huang et al.). Among the non pharmacologic approaches, acupuncture has been studied in primary dysmenorrhea and its efficacy can be predicted by pain-related functional connectivity patterns through machine learning multivariate pattern analysis (Yu et al.); the endocannabinoid system seems to be implicated in electroacupuncture-induced analgesia (MacDonald and Chen). Hence, the use of novel technological tools, e.g., machine learning, can improve the comprehension of the machinery of chronic pain. A better understanding of the above mechanisms can lead to discover the properties of already known and widely used analgesics, like acetaminophen, which still holds some surprises, e.g., the action of its metabolite N-acylphenolamine on brain TRPV1 and cannabinoid 1 receptors with consequent central analgesia (Ohashi and Kohno). These findings highlight the need for more basic as well as clinical studies on commonly used painkillers. One of the most remarkable is the case of opioids, often inappropriately used in fragile populations of the elderly commonly suffering from diseases that impair their communication skills. The treatment of chronic pain *per se* represents an area of strong therapeutic inappropriateness resulting in real-world limited access to care for fragile patients, e.g., demented aged people (Scuteri et al., 2017; Scuteri et al., 2018b; Scuteri et al., 2021). This is mirrored by the scarce use of the existing adequate observational pain scales and by the lack of standardization and guidelines for clinical practice in dementia (Gimenez-Llort et al.). This issue is very serious also for patients suffering from post-stroke pain. In fact, what emerges from the systematic review and meta-analysis of current literature is that the use of opioids in these patients is not specifically studied in the latter population with clinical trials designed for this purpose and using suitable pain assessment tools (Scuteri et al.). The assessment and the treatment of pain after spinal cord injury is still an unmet need (Finnerup, 2013; Yasko and Mains, 2018). The correct management of pain in patients who have been subjected to a severe brain injury with disorders of consciousness is fundamental to allow the best therapeutic approach and the planning of the most effective rehabilitation strategy (Riganello et al.).

## Data Availability

All the datasets generated/analyzed for this study are included in the article.
